# Hitherto unseen survival in an ALK‐positive‐patient with advanced stage adult ganglioneuroblastoma treated with personalized medicine

**DOI:** 10.1002/ccr3.1262

**Published:** 2017-11-07

**Authors:** Signe Risum, Ulrich Knigge, Seppo W. Langer

**Affiliations:** ^1^ Department of Oncology ENETS Neuroendocrine Tumor Centre of Excellence Rigshospitalet, Copenhagen University Hospital Copenhagen DK‐2100 Denmark; ^2^ Department of Endocrinology ENETS Neuroendocrine Tumor Centre of Excellence Rigshospitalet, Copenhagen University Hospital Copenhagen DK‐2100 Denmark

**Keywords:** Ganglioneuroblastoma, multimodal treatment, stage 4, survival

## Abstract

Survival of stage 4 ganglioneuroblastoma (GNB) patients is poor; no reports exist of patients surviving up to 5 years (1, 2). We report the clinical and therapeutic course of a patient with stage 4 GNB surviving beyond expectations due to a multimodal treatment approach incorporating new technologies in cancer diagnostic and treatment.

## Case

In November 2009, a previously healthy 34‐year‐old man presented with abdominal pain.

A CT scan showed a 4‐ × 7‐cm tumor located in the retroperitoneum. Preoperative blood samples and bone marrow aspirates were normal.

Surgery was performed; the tumor was growing into the wall of vena cava and could not be radically resected.

The histopathological report described a solid tumor, 8 × 5 cm, with encapsulated proliferation of atypical ganglion cells interspersed between a fibrillary matrix. The cells were immunoreactive for synaptophysin, chromogranin A, FLI‐1, and CD56. Fifteen percent of the cells were proliferating according to KI67. No amplification of myc‐n, 22q12, 1p, or 17q was found. The findings were consistent with a ganglioneuroblastoma but with some cells more resembling neuroblastoma.

Postoperative 18‐fluorodeoxyglucose positron emission tomography/computed tomography (FDG‐PET/CT) showed persisting tumor in the tumor bed and a small malignant process with high FDG uptake located in the spine at the 12th thoracic vertebra.

From January 2010 to June 2010, the patients underwent chemotherapy with cyclophosphamide, vincristine, and doxorubicin, alternating with carboplatin and etoposide. After four series, radiotherapy consisting of 60 Gy in 30 fractions was delivered to the tumor bed and to the spinal tumor.

In October 2011, recurrence consisting of four localized bone metastases to the pelvis and spine was detected on FDG‐PET/CT. The patient was treated with another four series of chemotherapy consisting of cyclophosphamide, vincristine, and doxorubicin, alternating with carboplatin and etoposide. Additionally, radiation therapy with 54 Gy in 18 fractions was delivered to the bone metastases.

Additional fluorescence in situ hybridization analysis (FISH) of the initial tumor specimen revealed an activating anaplastic lymphoma kinase (ALK) mutation F1174L in all tumor cells.

In November 2012, the patient developed progressive bone metastases and he started treatment with the ALK inhibitor crizotinib. In January 2014, FDG‐PET/CT showed complete remission of all lesions.

In July 2014, the bone metastases in the spine progressed again. ^68^Ga‐DOTATOC PET/CT showed grade 4 uptake in the metastases; the patient stopped treatment with crizotinib and was referred to peptide receptor radionuclide therapy (PRRT) with ^177^Lu‐DOTATATE given four times in intervals of 2 months.

In June 2015, ^68^Ga‐DOTATOC PET/CT revealed new bone lesions.

The original tumor specimen underwent further analysis for mutations in 50 genes using next‐generation sequencing (NGS) with Ion AmpliSeq Cancer Hotspot Panel v2 and AmpliSeq Colon Lung Cancer Hotspot Panel v2.

The NGS analysis revealed an activating epidermal growth factor receptor (EGFR)‐mutation L861Q in the tumor cells resembling neuroblastoma; the patient started treatment with the EGFR inhibitor erlotinib.

In January 2016, FDG‐PET/CT showed multiple new bone metastases; the patient started treatment with the second‐generation ALK inhibitor ceritinib.

As of April 2016, the patient is still in an excellent clinical condition, working full time as a high school teacher and responding to treatment.

## Discussion

This case describes the clinical and therapeutic course of an adult patient with stage IV GNB who is still alive 7 years after diagnosis and who continues to respond to treatment. Previously, no patients with stage IV GNB have been reported to live beyond 5 years after diagnosis 3, 4.

The negative prognostic factors in GNB are age at diagnosis (children <1 year old have a more favorable prognosis), advanced stage, primary tumor located in the retroperitoneum or in the adrenal glands, and tumor presenting with amplification of N‐MYC and deletion of the short arm of chromosome 1 5, 6.

The presented patient had all these prognostic factors except for amplification of N‐MYC and deletion of the short arm of chromosome.

Traditionally, treatment strategies incorporate surgery, radiotherapy, and chemotherapy based upon guidelines derived from pediatric experience.

In pediatric populations, patients have responded to first‐line chemotherapy with cyclophosphamide, vincristine and doxorubicin, and combinations with platinum and etoposide 7. For relapsed disease, topotecan and temozolomide have shown effect 8, 9. Over the last decade, tyrosine kinase inhibitors (TKIs) have been developed to target specific pathways associated with tumor growth. In lung cancer patients, inhibition of the EGFR and inhibition of the echinoderm microtubule‐associated protein‐like 4 ALK fusion oncogene are well‐known therapies for patients with specific mutations in these genes 10. The effect of TKIs in metastatic GNB is not clear.

In neuroblastomas, EGFR mutations have been reported very rare, and ALK mutations have been reported to occur in 8% of tumors 11, 12.

In general, detection of gene mutations and identification of patient subgroups that will benefit from a particular drug have improved with the use of NGS analysis 13.

The tumor specimen of the presented patient had an ALK mutation (F1174L) in all the tumor cells examined by FISH analysis; using NGS analysis, an additional EGFR‐mutation (L861Q) was found in the less differentiated part of the tumor resembling neuroblastoma. Both mutations were successfully targeted with TKIs resulting in clinical response.

In neuroendocrine tumors (NETs), somatostatin receptors are also used for targeted therapy in PRRT and for imaging. In imaging, the ability to tag somatostatin analogs with ^68^Ga has revolutionized the role of PET in diagnosis, staging, and therapy monitoring of patients with receptor‐positive NETs.

In PRRT, somatostatin receptors are targeted with radiolabeled peptides, thereby delivering a high radiation dose specifically to the tumor. The amount of tumor somatostatin receptors correlates with the efficacy of PRRT. In patients with a grade 3 or 4 uptake of somatostatin analogs, PRRT with any of the known radiolabeled somatostatin analogs such as ^111^In‐DTPA octreotide, ^90^Y‐DOTATOC, or ^177^Lu‐DOTATATE can result in symptomatic improvement 14.

Adverse effects of PRRT are few and mostly mild 15. Serious, delayed adverse effects such as myelodysplastic syndrome, renal failure, or leukemia are rare. However, loss of kidney function can occur after PRRT, with a decrease in creatinine clearance of about 4% per year for ^177^Lu‐DOTATATE 16.

Our patient was treated four times with ^177^Lu‐DOTATATE during 8 months; he has had no decrease in kidney function or bone marrow toxicity, and the bone metastases were stabilized.

In conclusion, this case underscores the importance of adapting a multimodal, therapeutic approach in patients with rare tumors such as GNB. By incorporating new technologies into cancer diagnostic and treatment, patients with otherwise limited therapy options could be offered treatment resulting in a possible improvement in survival rates. The potential adverse effects of increasing treatment intensity are a concern. In this case, however, side‐effects were limited; the patient is working full time as a high school teacher, is responding to treatment, and is alive 7 years after being diagnosed with stage IV GNB.

## Authorship

SR and SWL: acquired the data. SR, UL, and SWL: contributed with ideas, drafted the manuscript, and critically revised the manuscript.

## Conflict of Interest

None declared.
